# Recent Developments of Electrochemical Promotion of Catalysis in the Techniques of DeNOx

**DOI:** 10.1155/2013/463160

**Published:** 2013-07-16

**Authors:** Xiaolong Tang, Xianmang Xu, Honghong Yi, Chen Chen, Chuan Wang

**Affiliations:** ^1^College of Environmental Science and Engineering, Kunming University of Science & Technology, Kunming 650093, China; ^2^Civil and Environmental Engineering School, University of Science and Technology Beijing, Beijing 100083, China

## Abstract

Electrochemical promotion of catalysis reactions (EPOC) is one of the most significant discoveries in the field of catalytic and environmental protection. The work presented in this paper focuses on the aspects of reaction mechanism, influencing factors, and recent positive results. It has been shown with more than 80 different catalytic systems that the catalytic activity and selectivity of conductive catalysts deposited on solid electrolytes can be altered in the last 30 years. The active ingredient of catalyst can be activated by applying constant voltage or constant current to the catalysts/electrolyte interface. The effect of EPOC can improve greatly the conversion rate of NOx. And it can also improve the lifetime of catalyst by inhibiting its poisoning.

## 1. Introduction

In the 1970s, it has been shown that solid electrolyte played an important role in the heterogeneous catalytic. And the authors provided a measurement method of the oxidation degree of catalyst surface with the solid electrolyte doped ZrO_2_ [1]. In the late 1970s, Vayenas and Saltsburg [2] provided a concept of solid electrolyte potentiometry (SEP) which was used widely in the research of catalysis reaction mechanism on metal surface. These studies were helpful for the providing of EPOC. The phenomenon of EPOC was firstly reported by the group of Stoukides and Vayenas [3] at MIT in 1981. As a result, the actual enhancement of the catalytic activity was much higher than that estimated by the Faraday law. They called this phenomenon nonfaradaic electrochemical modification of catalytic activity (NEMCA). In the later research, scholars called it for EPOC.

In the last 30 years, it has been reported with more than 80 different catalytic systems that the catalytic selectivity and activity of catalytic active ingredient deposited on solid electrolyte can be altered greatly by applying constant voltage or constant current to the catalyst/electrolyte interface. The induced steady state conversion rate can be up to 150 times higher than the normal catalytic rate (open circuit) [4]. And it can be up to 3 × 10^5^ higher than the steady state rate of ion supply [5]. Because of the tightening legislation related to exhaust emissions, the removal of flue gas from exhaust duct has become increasingly important. As one of the main pollutants, NOx originating from automotive traffic and industries, especially in urban areas, has been a research emphasis. The traditional technology of three-way catalytic is ineffective for the removal of NOx under lean-burn conditions. And the normal techniques of DeNOx face three challenges. The most important one is to reduce or even remove the use of noble metals (Au, Pt, Pd, Rh, Ag, etc.) because of their excessive cost which makes necessary stages of recycling and recovery. Another point is to improve the lifetime of the catalysts by inhibiting their poisoning under operating conditions. The last crucial point is how to reduce the operating temperature. Thus, a new type of catalytic system for effectively cleaning the flue gas under lean-burn conditions is urgently needed. As one of the novel technology in the field of catalytic, the EPOC has some advantages compared with the traditional catalytic. Different obvious advantages of EPOC will be described such as the promotion of the catalytic activity and selectivity at low temperature, the improvement of the catalytic lifetime, and the enhancement of controllability. Most of the literature of EPOC were was reported for the study of catalytic application and the modification of catalysts. However, there is not a recapitulative article only for electrochemical promoted DeNOx. The objective of this paper is to make the overview for the special performance of EPOC at different influencing factors and its potentialities in the field of De-NOx by describing different examples.

## 2. Reaction Mechanism

**Figure 1:**
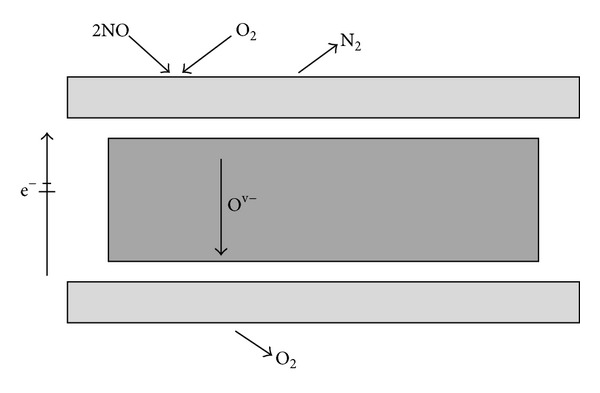
Catalyst structure schematic drawing.

The term EPOC was used to describe the phenomenon that the pronounced strongly nonfaradaic and the reversible changes in the catalytic activity and selectivity of conductive catalysts deposited on solid electrolytes. Vayenas et al. [6] have provided a theoretical basis and a detailed account of the phenomenology, which made the EPOC clear. The operating process usually requires electrochemical pumping of ions to the interface of porous working electrode and solid electrolyte. As a result, the modification in work function changed the activation energy of reactions and the adsorption enthalpy of adsorbed species. Controlling voltage provides control of the concentration of spilt over promoter species on the solid electrolyte surface. Therefore, the metal electrode, as catalytically active locus, is usually in the form of an electronically conducting and porous metal plate placed on the solid electrolyte. Catalyst structure schematic drawing is outlined in Figure 1.

The idea of reducing NOx with electrochemical method in a solid-state cell was firstly suggested by Pancharatnam et al. in 1975 [7]. The authors suggested that the reduction of NO occurred on the zirconia surface, even interface between electrode and solid electrolyte, and not on the electrode itself. The NOx received e^−^ and was vented in the form of gaseous nitrogen to air. The O^v−^ was transferred through the solid electrolyte to metal anode plate and vented in the form of O_2_. Researches made it clear that this process was more facile reaction under oxidizing conditions [8]. The cathode had a high activity and selectivity towards the reduction of NOx when O_2_ was present along with NOx. The result showed that the reduction rate of NOx exceeded that estimated with the Faraday law by a thousand fold under a high cathodic overpotential condition. Taking that into account, the mechanism below was proposed by the authors:

(1) Vo••(s)⟷Vox(s)+2helectrode•NO+Vo••(s)→N−Oox(s)NO+N−Oox(s)→N2O+Oox(s)N2O+Vox(s)→N2+Oox(s)Oox(s)+Vo••⟷Vo••(s)+Oox(b),

where V_*o*_^*x*^ is a F-center, (*s*) the surface of electrolyte, and (*b*) the bulk of the electrolyte. This hypothesis was authenticated by Gür and Huggins using Pt and Au point electrodes in the later work [9]. In a work by Gessner et al. [10] in 1988, the authors suggested that oxygen conversion was not always the dominant charge transfer reaction. The oxidation of nitric oxide to nitrogen dioxide and the reduction of nitrogen dioxide to nitric oxide were found to be the dominant charge transfer reactions in their work.

 Three parameters usually describe the magnitude of electrochemical promotion:

(1) the rate enhancement ratio (*ρ*) defined from

(2) $$\begin{array}{c} \rho =\frac{r}{r_{o}}, \end{array}$$

where *r* is the electropromoted catalytic rate, *r*_*o*_ the unpromoted catalytic rates,

(2) the faradaic efficiency (Λ) defined from

(3) $$\begin{array}{c} \Lambda =\frac{\Delta r}{\left(I/2F\right)}, \end{array}$$

where Δ*r* is the potential or current induced change in catalytic rate, *I* the applied current, *F* the Faraday's constant,

(3) the promotion index (PI_*j*_) of the back-spillover promoting species defined from

(4) $$\begin{array}{c} \text{P}{\text{I}}_{j}=\frac{\Delta r/r_{o}}{\theta_{j}}, \end{array}$$

where *θ*_*j*_ is the coverage rate of the promoting species (*j*) on the catalyst surface.

A reaction that exhibits electrocatalysis is limited to |Λ| ≤ 1, while electrochemical promotion when |Λ| > 1. A reaction is termed electrophilic when Λ < −1, while electrophobic when Λ > 1. In the former case, the rate decreases with catalytic potential, *U*, while in the latter case the rate increases with catalytic potential. Λ values up to 3 × 10^5^ [11] and *ρ* values up to 1 × 10^3^ [12] have been found for several systems.

**Figure 2:**
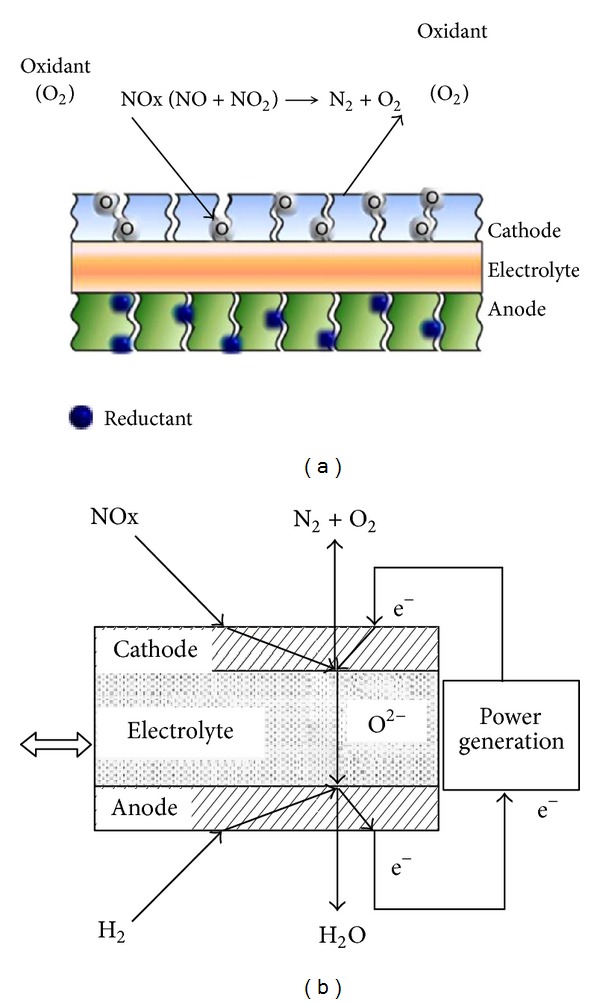
Schematic diagrams of electrochemical-catalytic DeNOx via (a) the ECC and (b) the SOFC.

 Solid oxide fuel cells (SOFCs) show good results for lean NOx emission control of high-concentration NOx with or without power generation in Ta-Jen Huang's research [13–18]. The simplified catalytic system, without consuming any reductant, that a new generation catalytic converter (electrochemical-catalytic cells, ECCs) can have a very compact size to be used for lean-burn motor vehicles [19–21]. Authors offered the transformation process of ions on the catalysis (shown in Figure 2) [22]. They think that either the SOFC or the ECC is by direct NO decomposition because there is no reductant over the cathode. The mechanism was suggested as below [22]:

(5) NO2→NO+ONO→N+O2N→N22O→O2.

Considering [] as the surface oxygen vacancy [23]

(6) NO+[]·[]→N−[O]·[]NO+N−[O]·[]→N−[O]·[O]−NN−[O]·[O]−N→N2+[O]·[O][O]·[O]→O2+[]·[],

where []·[] is a pair of adjacent surface oxygen vacancies and [O] denotes O in the surface oxygen vacancy,

When CO_2_ and H_2_O are present in this catalytic system [22]:

(7) CO2+[]→CO+[O]NO+∗−[]→N∗−[O]CO+N∗−[O]→CO2+N∗−[]H2O+[]→H2+[O]H2+N∗−[O]→H2O+N∗−[],

where ∗ denotes the active site for NO adsorption *via* N. The products are only one N species which should be distantly distributed. The surface diffusion of N species would influence the production of N_2_ in the low NOx concentration region. 

 When a reductant (e.g., C_3_H_6_) is used in this catalytic system [24],

(8) NO(g)→NO(ads)C3H6(g)→C3H6(ads)O2(g)→2O(ads)NO(ads)→N(ads)+O(ads)N(ads)+N(ads)→N2(g)N(ads)+NO(ads)→N2O(g)C3H6(ads)+9O(ads)→3CO2(g)+3H2O(g).

Comparing all the previous mechanism analysis, we may safely draw the conclusion that the electrochemical catalytic process can be divided into the following several steps: (I) the adsorption of reactant gas; (II) gas molecules combined with the active site; (III) the forced transfers of specific ions due to the effect of supplied voltage; (IV) the mutual restructuring of adsorbed ions, then desorption. The electrochemical catalytic efficiency is influenced by the steps (II) and (III), while the selectivity of products is related to the last step.

## 3. Influencing Factors

### 3.1. Types of Electrode and Solid Electrolyte

At the present, noble metals are used as electrode plate mainly including Au, Pt, Pd, Rh, Ag, and Ir [7, 9, 25–32]. There are special *d* electronic configurations in the outer electrons of these noble metals. This configuration provides coordination bond for the reactants on the catalyst/electrode interface. Reactants are activated by the coordination bond during the reaction process. Therefore, the noble metals materials have became the targets of preferred study. However, some scholars have begun to search for cheap materials to replace these noble metals because of their excessive cost which makes necessary stages of recycling and recovery.

The cheap metal oxides CuO and NiO were studied in some papers [33, 34]. The result showed that NiO has few activities towards the reduction of NO. Nevertheless, CuO is highly active towards the electrochemical reduction of NO. It is the same phenomenon as that observed for the traditional catalytic reduction of NO on these two oxides. And it is known that CuO has a better catalytic activity than NiO [35, 36]. These papers made it clear that the bond breaking of NO was one of the restrictions for the electrochemical reduction of NO. The result also showed that the reduction rate of NO was increased when NiO reduced to Ni metal, whereas the reduction rate of oxygen was restrained when Cu was presented as CuO. Yet the reduction of oxygen was induced when CuO was reduced to Cu_2_O.

**Figure 3:**
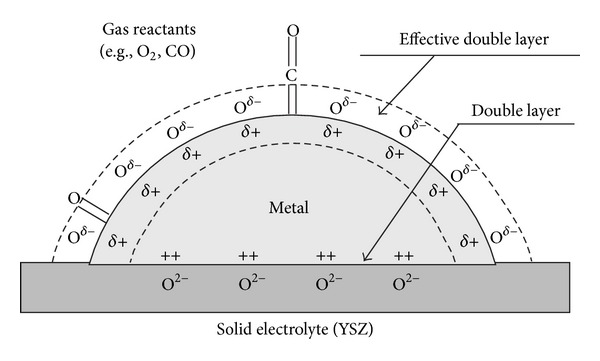
Elementary diagram representation of a metal electrode deposited on a O^2−^ conducting solid electrolyte.

Furthermore, other scholars have done a lot of work towards solid electrolyte. It was shown that the solid electrolyte is indispensable in the process of heterogeneous electrochemical catalysis reduction of NOx. The working principle of solid electrolyte in the process of EPOC has been expounded in reaction mechanism (Section 2). It will be expounded with an example in the next moment. For the solid electrolyte of O^2−^ conductor supports, the action of EPOC has been found to derive from the anionic transfer (reverse spillover) of O^*δ*−^ species (Figure 3) [37]. These O^*δ*−^ species together with their image charge in the metal formed a whole neutral double layer at the metal-gas interface. Both chemisorptions and catalysis are affected by the back-spillover and the image charge in a pronounced manner. The back spillover O^*δ*−^ species are different from oxygen adsorbed from the gas phase under a high oxygen condition [38–40]. They are also less against catalytic oxidations than gas supplied oxygen.

The EPOC effect has been confirmed for a wide field of reactions in the reduction of NOx when the electrodes are connected to a solid electrolyte. Electrochemical promotion was also observed on oxides such as CuO [33], RuO_2_ [41], and IrO_2_ [42]. Several parts of literature [43–47] have reported that the using the class of oxides, the based electrodes were spinels, as cathodes for the electrochemical reduction of NOx. A research result reported by Wachsman et al. [48] manifested that an La_0.8_Sr_0.2_Co_0.9_Ru_0.1_O_3−*δ*_ cathode had a higher conversion rate in reducing NOx than a Pt-based cathode under oxidizing conditions. This phenomenon was attributed to the good electrochemical catalytic characters of the perovskite-based cathode compared to the Pt-based cathode. In this kind of study, the solid electrolyte may be mixed ionic-electronic conductors (CeO_2 _[49] and TiO_2 _[50]), F^−^ conductors (CaF_2_) [51], O^2−^ conductors (YSZ (Y_2_O_3_-stabilized ZrO_2_)) [52–54], H^+^ conductors (Nafion [55] or CaZr_0.9_In_0.1_O_3−*α*_ [56, 57]), and Na^+^ conductors (*β* ^*″*^-Al_2_O_3_ [58, 59] or Na_3_Zr_2_Si_2_PO_12_ [60]). It is also a good research field that coating of metal based electrodes.

### 3.2. Configurations of Electrochemical Promotion Reactors

**Figure 4:**
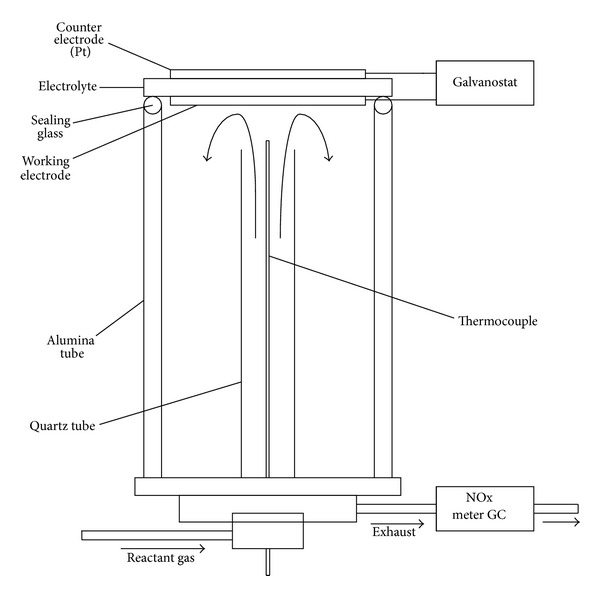
Configuration of reactor that only working electrode is exposed in the reactant gas.

**Figure 5:**
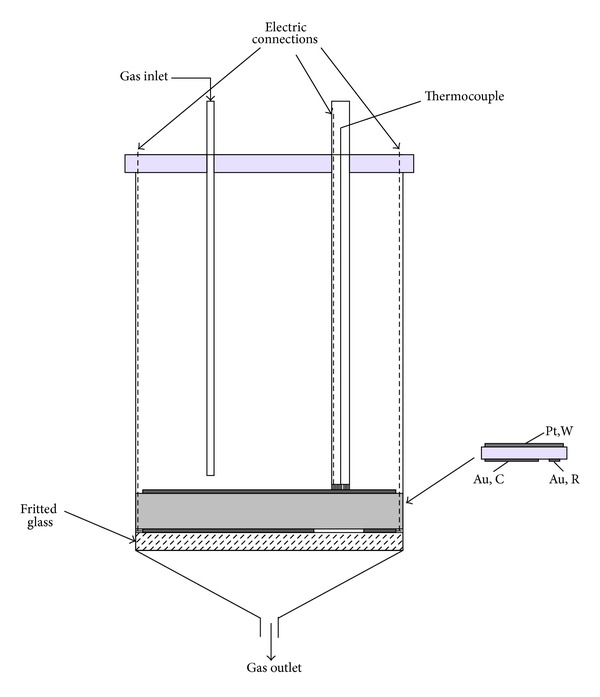
Configuration of reactor that both electrodes and solid electrolyte were exposed in reactant gas.

Electrochemical promotion has been studied for over eighty different catalytic systems [37], while it has mainly two kinds of forms of electrochemical promotion reactors classified in structures. One is to which the reactor only working electrode is exposed in the reactant gas (Figure 4). The other is the reactor that both electrodes and solid electrolyte were exposed in reactant gas (Figure 5). The porous catalyst film or working electrode of the reactor shown in Figure 4 is exposed in gas mixture, while counter electrode is exposed in air. Because the process of EPOC just occurred on the surface area of the film, the reactor shown in Figure 4 has more advantages compared with the reactor shown in Figure 5 in the mechanism research of EPOC. In the paper by Song et al. [61], the cathode was assembled in a bilayer structure. The Pt paste was screen-printed as a circle on the yttria stabilized zirconia (YSZ) disk. The anode was also the platinum applied on the other side of the solid electrolyte. Then, metal lines were contacted with the platinum layers at both sides. In the consideration of impedance measurements, the platinum reference electrode applied on the opposite side of the working electrode was used. The result showed that the reduction behavior of NO presents a function of the applied current for the electrochemical promotion cells. It required a threshold current to arouse the EPOC behavior of NO for all the cells. The EPOC behavior of NO did not occur if external current was applied insufficiently. Its conversion rate was increased abruptly when a higher current density was supplied to the cell. It is feasible that the conversion rate of NO reach to 87% at 250 mA/cm^2^, although this reaction started at 60 mA/cm^2^.

In contrast, all the counter electrode, working electrode, and solid electrolyte of the reactor shown in Figure 5 are exposed in the gas mixture. The catalytic active sites spread over carrier contact easily with gas mixture compared with another one. Based on the previous advantage, the reactor shown in Figure 5 has a higher value in the EPOC applied research. In the paper by Dorado et al. [24], the EPOC reactor was made of a pyrex tube. And the catalytic experiments were operated at atmospheric pressure. The catalytic reaction process was carried out in this kind of tubular solid electrolyte reactor. The temperature of catalytic reaction was detected with a K-type thermocouple installed inside the inner of reactor. And the external heat producer of the EPOC reaction was a furnace outfitted with a heat control system. The result showed that the system in catalytic potential modification on reaction rates could be electrochemically promoted. The conversion rate of NO was increased at low O_2_ concentration (0.5 and 1%) under the traditional optimally promoted conditions. However, it could be seen that the conversion rate of NO was decreased when the O_2_ concentration increased, eventually resulting in an entire loss. The increase of O_2_ concentration results in a decreasing of the efficiency of EPOC for NO reduction. This phenomenon could be attributed to that a relative increase of the surface coverage of O_2_ and a strong inhibition of the reductant adsorption.

**Figure 6:**
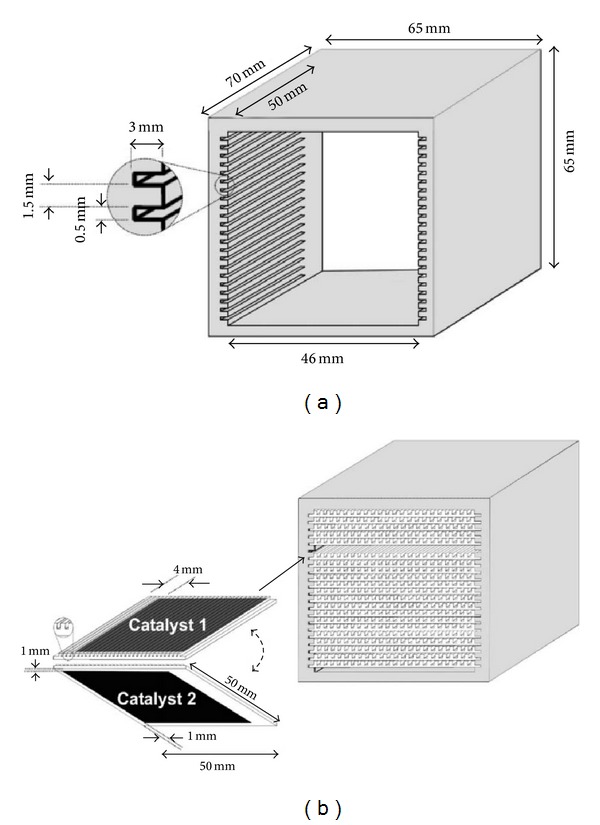
Schematic and dimensions of the monolithic electrochemical promoted catalysis reactor.

Although the EPOC has been studied for more than 30 years, there has been no large-scale commercial operation. It is chiefly because of the lack of compact and efficient reactor designs allowing for the operation of EPOC. In the paper by Balomenou et al. [62], a novel dismountable monolithic-type EPOC reactor and an ingenious sensor-catalytic reactor unit have been designed and tested for the reduction of NO by C_2_H_4_ under an oxygenic condition (Figure 6).

The reactor can be considered as a complex between a ribbed-plate or flat-plate solid oxide fuel cell and a traditional monolithic honeycomb reactor. Two external electrical connections were required in this novel reactor. And the novel reactor achieves easily practical utilization of the EPOC. In this novel reactor, thin (about 20 ~ 40 nm) porous catalyst films were made of two different materials (Au and Rh, Pt and Rh). These films are sputter-deposited on the opposing surfaces of solid electrolyte. The shapes of solid electrolyte are thin (0.25 mm) parallel plates. And the solid electrolyte parallel plates were supported in the grooves of a ceramic monolithic holder. The Au/YSZ/Rh-type serve as sensor elements and the Pt/YSZ/Rh-type as electrochemical promoted catalyst elements. The 22-plate reactor was tested under high flow rate (1.8 L/min) and gas hourly space velocity (1200 h^−1^) condition. This novel reactor could achieve higher conversions (about 90%) than all former electrochemical promoted catalysis units and showed significant promise for the commercialization and practical applications of EPOC.

### 3.3. With or without Reductant

The EPOC can be divided into two categories according to whether the reductant is consumed. The unsaturated hydrocarbon compounds (HC) is usually used as reductant. Notably, propylene is usually used to represent HC in the engine exhaust [63]. The selective catalytic reduction of NO by C_3_H_6_ was investigated by Constantinou et al. [27]. His group studied the effect of EPOC on porous polycrystalline Rh catalyst-electrode films. The result showed that the rate of NO reduction and CO_2_ formation was enhanced, respectively, by factors of up to 55 and 200 due to the application of current or potential between the Rh catalyst-electrode and an Au counter electrode.

**Figure 7:**
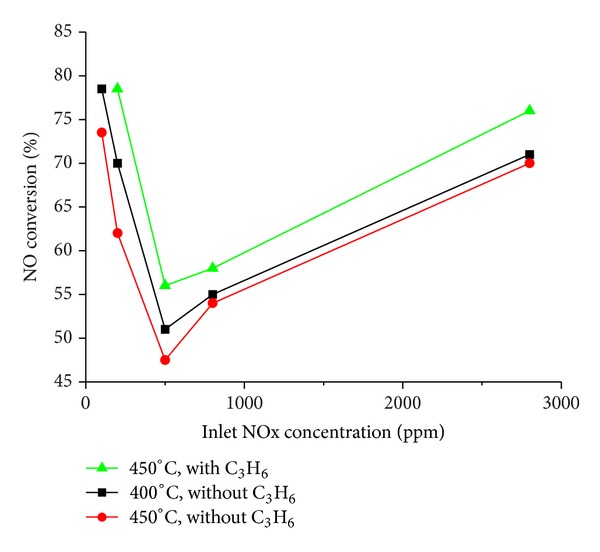
Effect of NOx concentration, temperature and propylene on NO conversion.at 400 and 450°C with or without 350 ppm C_3_H_6_; other component was 25 ppm SO_2_.

Huang et al. [64] attempt to clear simultaneously NOx and hydrocarbons with electrochemical catalytic. The result showed that a higher oxygen concentration is beneficial to both the NO conversion and the hydrocarbons oxidation to result in zero pollution. The effect of adding propylene for NO removal was also investigated (result shown in Figure 7). Figure 7 shows that both adding propylene and decreasing temperature increase the NO removal. Moreover, The effect of decreasing temperature from 450 to 400°C is smaller than that of adding 350 ppm propylene. The effect of adding propylene is similar to that of HC-DeNOx over catalyst [65]. NOx can be reduced by propylene. Besides, C_3_H_6 _[66–68], many other species of HC (including CH_4_, C_2_H_4_, C_3_H_8_, and C_5_H_12_) [69, 70] were investigated. The results showed that the presence of HC is favorable for NOx removal. This electrochemical promotion is also present at the catalytic system that CO [71] or NH_3_ [72] is reductant. But the presence of reductant may inhibit other forms promotion in same times.

In the paper reported by Dorado et al. [24], the effect of EPOC for the reduction of NO by C_3_H_6_ was studied. This effect was firstly investigated on a Pt impregnated catalyst film directly deposited onto an Na-*β* ^*″*^-Al_2_O_3_ solid electrolyte. The result showed that the presence of promoters enhanced the selectivity of N_2_. However, combined with characterization results, the promotional effect of sodium on the overall catalytic activity for NO removal would be inhibited when C_3_H_6_ and O_2_ are present. Authors thought that the phenomenon can be attributable to the result of a strong inhibition of C_3_H_6_ adsorption and a relative increase of the O_2_ coverage.

The electrochemical promotion of decomposition is an effective method for NOx removal. In 2001, the electrochemical cells of oxide*|*Pt (cathode*|*YSZ*|*Pt (anode) for NO decomposition were designed and investigated [73]. It was shown that the properties of the electrochemical cell for NO decomposition and the value of the current efficiency could be enhanced because of the specific microstructure of the NiO-YSZ mixed oxide. And an electrochemical cell for NO decomposition was firstly designed for which the value of current efficiency is exactly equal to the theoretical one. In the following studies, his group proved that the NO conversion was positively associated with the value of the current, while the value of current efficiency is only dependent on the NO and O_2_ gas concentrations [74, 75]. It is possible to minimize the values of the cell operating voltage by the control of the composition of the (NiO)_*x*_-(YSZ)_1−*x*_ electrocatalytic electrode [76]. In 2004, his group proposed a novel electrochemical promotion reactor for NOx decomposition. This reactor was designed by compositional control and nanostructural of an NiO-YSZ electrochemical promotion catalytic electrode [77]. In such reactors, the electrical power required for NO decomposition is greatly reduced in the presence of 10% of O_2_. Therefore, the energy consumption required for NO removal in such reactor is lower than that in traditional cells. 

The catalytic activity of electrochemical promotion decomposition for NOx was strongly influenced by microstructure, composition, and the configuration of the working electrode [78, 79]. The cell composed of (La_2_Sn_2_O_7_ + YSZ)/Pt composite electrode was investigated by Park et al. [79]. A higher catalytic activity of electrochemical promotion decomposition was observed for the cell composed of (La_2_Sn_2_O_7_ + YSZ)/Pt composite electrode than the Pt electrode. The result showed that 87% NOx was reduced at the current density of 194 mA/cm^2^ in the reactant gas containing 2% O_2_, while such cell decomposed 80.5% NOx at the current density of 325 mA/cm^2^ under 4% O_2_ condition. The cell stacks composed of Ce_0.9_Gd_0.1_O_1.95_ porous electrolyte and La_1−*x*_Sr_*x*_MnO_3_ (*x* = 0.15, and 0.5) composite electrode were investigated by Werchmeister et al. [80]. The cell stacks were infiltrated with the nanoparticles of Ce_0.9_Gd_0.1_O_1.95_, Ce_0.8_Pr_0.2_O_2−*δ*_ and pure ceria after sintering. It is possible to reduce up to 35% of NO present when the cell stacks are polarized with 1.5 V for each cell. It is shown that the cell stacks infiltrated with pure ceria had the highest electrochemical catalytic activity. However, the highest selectivity towards NO compared to O_2_ present at the ones infiltrated with Ce_0.9_Gd_0.1_O_1.95_.

The electrochemical promotion of catalytic deoxidation and decomposition is an effective way to NOx removal. The EPOC deoxidation for NOx usually has a higher NOx conversion due to the presence of reductant. However, the reductant should cause secondary pollution if the catalytic process is an incomplete reaction. The proportion of added reductant should be paid enough attention. The EPOC decomposition for NOx is an ingenious way to avoid the pollution caused by reductant. However, compared with deoxidation, the conversion of NOx of the EPOC decomposition is unsatisfactory. Therefore, improving the NOx conversion of the EPOC decomposition would become a direction with quite development potentiality in the future. 

## 4. Recent Positive Results

In 1990, Cicero and Jarr [81] reported firstly the use of oxide-based electrodes in the reduction of NO. The authors used a metal oxide-based cathode to remove NO, which achieved a conversion of 91% with O_2_ concentration of 8%. The temperature range of experiment was from 650°C to 1050°C. But they did not give the magnitude of the current efficiency in this paper. The influence of NO for the reduction rate of O_2_ on La_0.8_Sr_0.2_MnO_3−*δ*_ based electrodes was reported in 1995 [82]. Reinhardt et al. found that the reduction rate of O_2_ was increased when NO was added to the gas mixture in the temperature range of 500 ~ 900°C. But they did not undertake the gas analysis when NO was added to the gas mixture. Therefore, it is possible that the reduction of NO itself led to the current density increased.

In 1996, Palermo et al. [83] did a deeper research on the system used either propene or CO as reductant. The result showed that an obvious increase of NO reduction rate was achieved when Na^+^ were pumped to the catalysts surface. The authors made a point that the increase of NO reduction rate was related to operated temperature, applied potential, and gas composition. The maximum increase of NO reduction rate was achieved at 375°C when a low potential (0.25 V) was applied on the system. Authors thought the Na^+^ could induce weakening of the NO bond, which led to a easier dissociation of NO bond. This step played an important role in the enhancement of NO reduction rate. In the later research, Yentekakis et al. [84] found that the reduction of NO with propene was observably enhanced when Na^+^ was pumped to the Pt surface. In conclusion, authors thought this enhancement in the reduction of NO owed to a sodium-induced promotion of the NO bond dissociation.

 In 1997, a paper reported by Marina et al. [85] narrated the reduction of NO with H_2_ using a Pt catalyst on *β* ^*″*^-Al_2_O_3_ Na^+^ conducting solid electrolyte. The research showed that the electrochemical promoted catalytic reduction rate of NO was increased up to 30 times more than the unpromoted catalytic rate. What is more, the electrochemical promoted catalytic reduction rate of NO was increased with over thousands times more than the rate of Na^+^ pumped to the catalysts surface. At the same time, the catalytic selectivity of NO to N_2_ was increased from 30% to 75%. In 1999, a research reported by Belyaev et al. [86] investigated the electrochemical promoted reduction of NO with CO. In this research, authors used Pt material as catalysts supported on YSZ. The result showed that the reduction rate of NO was strongly increased when the current was applied to cathodic.

In the research published in 2000, Kaneko et al. [87] found that NO could be reduced at 800°C after being injected in pulses. Authors used a platinum electrode placed on the YSZ and provided relatively high potentials (−500 mV versus air) to the system. In the study by Hibino et al. [88], it was shown that the alternating current efficiency was highest when the applied potentials were higher than 3 V in combination with the use Pd electrode. However, the direct current efficiency was highest when the applied potentials of lower than 3 V. 

In 2001, Bredikhin et al. [73] attempted to use a multideck electrode structure. The multideck electrode structure consisted of an NiO/YSZ electropromoted catalytic active layer, a YSZ covering layer and a Pt/YSZ cathode. The result showed that the activity of the cathode layer was related to the Pt/YSZ ratio. 

 In 2003, a paper reported by Vernoux et al. [89] narrated that the platinum was supported on NASICON which was a kind of Na^+^ conducting electrolyte. And the propene was used as reductant for the reduction of NO. The result showed that the reduction rate and the selectivity of NO to N_2_ was increased when a low potential (100 mV) was applied on the system. It is possible that nitric oxide was efficiently reduced at low temperature of 300°C. The use of the NASICON electrolyte made it possible that the electrochemical promoted catalysis reaction was operated at a low temperature. In the research by Petrushina et al. [90], a proton conducting H_3_PO_4_ based electrolyte was used to the reduction of NO at a lower temperature (135°C). The H_2_ was used as reductant in this electrochemical promoted catalysis system. The result showed that the reduction rate of NO could be enhanced when the Pt electrode was provided a negative potential.

In 2005, Kammer and Skou [91] studied the Fe-Mn-based perovskites catalyst. From their research, the result showed that the Fe-rich perovskites had the highest catalytic activity in the reaction of the electrochemical promoted reduction of NO. This result identified with the hypothesis that the reduction rate of NO was determined by the amount of oxide ion vacancies and the redox capacity. However, in another paper by Simonsen et al. [92], the catalytic activity was decreased after adding BaO to the perovskites-based electrode. In this research, the catalytic selectivity was also investigated. In the conclusion, the authors presented that the selectivity was strongly enhanced after adding BaO to the perovskites-based electrode.

In 2006, the influence of the YSZ covering layer was studied again by Hamamoto et al. [93]. The result showed that the YSZ covering layer led to the suppression of the adsorption and the decomposition of O_2_. In 2008, the multideck electrode structure was studied by the same group of Hamamoto et al. [94]. In this research, the top of the multideck electrodes applied an extra-covering layer. This covering layer consisted of Na, K, or Cs together with Pt and Al_2_O_3_, which were used as NOx adsorbing layer. At last, it was shown that the adsorbing layer containing K appeared a better effect than others. This type of cathode in the paper could achieve a quite high catalytic activity. And it is possible that the conversion of NOx is increased about 20% due to the current effect. Therefore, this type of multideck electrodes is a developed direction in the research of removing NOx.

The effect of EPOC can be used to activate a metal catalyst for the selective catalytic reduction of NOx under wet reaction conditions. In 2009, the effect of some operating conditions on the simultaneous removal of NOx and SO_2_ was investigated. The simulated NO-SO_2_-air flue-gas mixtures were bubbled into a integrated wet scrubber electrochemical cell system in Il-Shik Moon's research [95]. The result showed that the NOx was fast and greatly reduced when SO_2_ coexisted in the scrubber column. And it was proved that the SO_2_ removal from the NO-SO_2_ mixture occurred independent of NOx with no interference what so ever. In the paper reported by de Lucas-Consuegra [96], the catalytic performance of Pt electrode can be optimized by the application of different potentials at each operation temperature. The catalytic behavior of the system is optimized due to the combined use of the Pt/K-*β*Al_2_O_3_ cell under changing reaction conditions.

The effect of voltage and temperature on NO removal with power generation in a solid oxide fuel cell (SOFC) unit was investigated in 2010 [97]. The SOFC is constructed with Ni-(Ce,Gd)O_2−*x*_ as anode, YSZ as electrolyte, and V_2_O_5_-added (LaSr)(CoFe)O_3_-Ni-(Ce,Gd)O_2−*x*_ as cathode. It is shown that the NO conversion increases slightly with the decreasing voltage but with increasing temperature from 800°C to 875°C. And the NO conversion increases as O_2_ and NO concentrations decreases when the process is operational under 2–5% O_2_ concentration condition.

 In the paper reported by Hadjar et al. [98], an electrochemical NOxTRAP catalyst Pt-Ba/YSZ was investigated. The NOxTRAP catalyst is one of the technology of DeNOx [99]. It is shown that the cathodic polarization is beneficial to the NOx storage even under lean-burn conditions. The experiment was operated at 500°C with different O_2_ partial pressures. The duration until full NOx storage was drastically enhanced about 80 times in the presence of 6% O_2_. And NOx can be reduced about 10% due to the occurrence of electrochemical reduction during regeneration phases. Authors thought that the generation of oxygen vacancies on the YSZ surface induced by negative polarization is the major influence factor related to the electrochemical activation of the NOx storage capacity.

An ingenious multilayer electrochemical cell was investigated in 2012 [100]. An ytrria stabilized zirconia cover layer was replaced with an adsorption layer of the cell. It is shown that the electrochemical properties of NOx removal were dramatically enhanced. Authors thought that the enhancing of the NOx removal was related to the following two aspects: the extensive release of selective reaction sites for NOx species, a strong promotion for NOx reduction as adsorption layer connected with both the Pt and catalytic layers. The optimizing of electrochemical cell may provide a promising direction for NOx emission control [101].

## 5. Conclusions

It has been shown that the catalytic activity and selectivity of a few catalytic reactions can be modified by electrochemical promotion. Many studies have been reported related to the effect of EPOC during the last 30 years. The study about its mechanism and application is becoming a trending topic in the field of reduction NOx. It is possible that the electrochemical promotion reduction of NOx was operated in a few types of solid-state electrochemical cells. It was reported that the cathode materials or catalysis species with an enough coordination bond were effective for the electrochemical promotion reduction of NOx. The importance of the EPOC phenomenon both in electrochemistry and catalysis was highlighted with the effectiveness of EPOC for catalytic oxidations and reductions using different types of catalysts, electrodes, and solid electrolytes. Further development of catalysts, electrodes, and solid electrolytes materials are needed in order to increase the reduction rate of NOx. The improving lifetime of the catalysts also appears quite promising. The development of large-scale novel monolithic applicable reactors with ingenious design may be beneficial to the practical utilizations of EPOC.
